# Synergistic Enhancement of Osimertinib Efficacy in Non-small Cell Lung Cancer Cells Through Epigallocatechin-3-Gallate: Mechanistic Insights Into YAP/TEAD/CTGF Axis Inhibition

**DOI:** 10.34172/apb.43809

**Published:** 2025-03-23

**Authors:** Ashwini Somayaji, Chakrakodi Shashidhara Shastry

**Affiliations:** ^1^Department of Pharmacology, Faculty of Pharmacy, M.S. Ramaiah University of Applied Sciences, New BEL Rd, M S R Nagar, Karnataka- 560054, India; ^2^Nitte Gulabi Shetty Memorial Institute of Pharmaceutical Sciences, Paneer, Karnataka, India

**Keywords:** Epigallocatechin-3-gallate, EGFR T790M mutation, Hippo pathway, Osimertinib

## Abstract

**Purpose:**

Combinatorial therapies are essential for treating advanced non-small cell lung cancer (NSCLC), particularly overcoming resistance to third-generation epidermal growth factor receptor (EGFR) like osimertinib (OSI). The Hippo signaling pathway, a critical regulator of cell proliferation, apoptosis, and tumor progression, is often dysregulated in NSCLC and contributes to chemo-resistance. This study investigated the potential of epigallocatechin-3-gallate (EGCG), a green tea polyphenol, to overcome OSI resistance by modulating the Hippo signaling pathway, specifically through inhibition of the YAP-1 (Yes-associated protein)-TEAD (TEA domain transcription factor)-CTGF (connective tissue growth factor) axis.

**Methods:**

Using stepwise dose escalation, OSI-resistant (OR) clones were developed from EGFR T790M-mutated H460 cells. The anti-proliferative effects of EGCG were assessed, and synergistic interactions between OSI and EGCG were analysed using combination index (CI) values and the median effect concept. Mechanistic studies evaluated the co-treatment’s impact on the Hippo signaling pathway, focusing on the inhibition of the YAP/TEAD/CTGF signaling axis.

**Results:**

The OR clones exhibited significantly higher IC_50_ values for OSI (25.12–28.48 µM) compared to parental H460 cells (2.74±0.2µM). EGCG treatment reduced cell viability in a concentration-dependent manner, with IC_50_ values of 102.54±0.23μM for H460 cells and 225.79–237.36 µM for OR clones. Combination treatment of OSI and EGCG showed strong synergy at a 1:2 molar ratio, with CI values indicating synergism across a range from IC_50_ to IC_95_. Mechanistically, co-treatment suppressed the overexpression of the YAP/TEAD/CTGF axis, restoring Hippo pathway activity and reversing OSI resistance.

**Conclusion:**

This study provides evidence that EGCG effectively targets the Hippo signaling pathway to overcome OSI resistance in NSCLC. The inclusion of EGCG in combinatorial therapies holds promise as a novel approach to combat therapeutic resistance and improve outcomes for patients with EGFR-mutated NSCLC.

## Introduction

 Cancer resistance poses a significant global challenge, often leading to unfavorable treatment outcomes and recurrence in patients, even after successful therapy. Non-small cell lung cancer (NSCLC) accounts for 80% of lung cancer cases. Despite its early detectability and the availability of modern therapeutic options, it remains a formidable disease. Targeted drug therapies have been developed to address the altered cell growth mechanisms in NSCLC.^1^ The treatment of advanced-stage NSCLC relies on immunotherapeutic drugs, particularly tyrosine kinase inhibitors (TKIs), which target specific mutations in the epidermal growth factor receptor (EGFR) gene. Several TKIs have been approved for NSCLC treatment and are classified into first-generation (e.g., gefitinib, erlotinib) and second-generation (e.g., afatinib) inhibitors. These drugs have shown significant clinical benefits in patients with advanced NSCLC harboring Ex19del and L858R mutations.^2,3^ Although initial EGFR TKI therapy provides good disease control, acquired resistance is pervasive and remains a major obstacle.^4^

**Figure 1:**
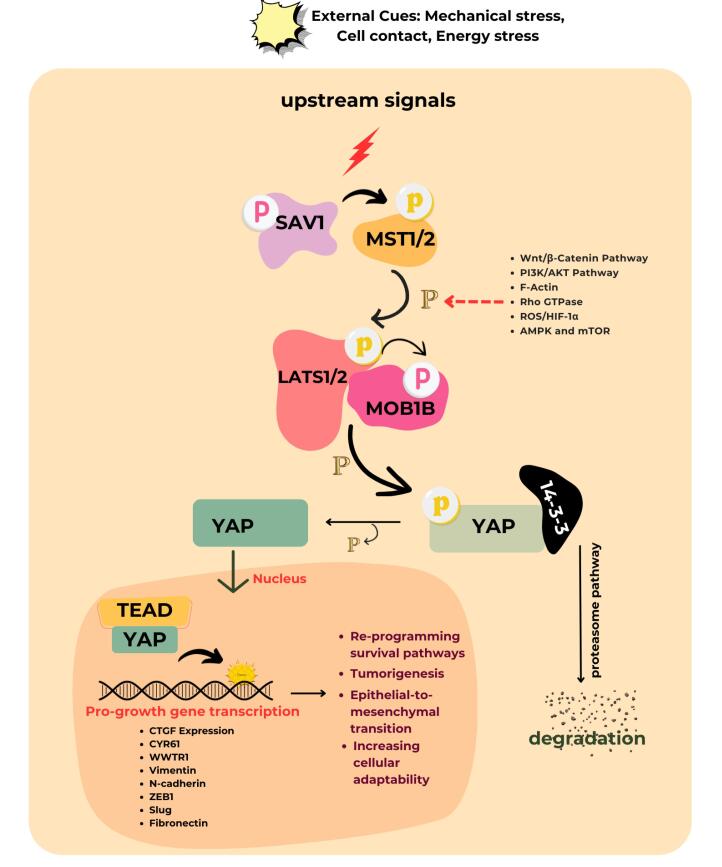
The canonical Hippo signaling pathway regulates YAP-1. When cells are in contact with each other, upstream signals are initiated that activate LATS1/2 kinases. This activation leads to the phosphorylation of YAP-1, causing their retention in the cytoplasm through binding to 14-3-3 proteins. Further phosphorylation by CK1 kinase targets them for degradation. In contrast, at low cell density, the MST and LATS complexes are inactive. Consequently, un-phosphorylated YAP-1 can enter the nucleus and bind to transcription factors such as TEAD factors. This binding results in the transcription of genes involved in cell growth, survival, cell fate, chemo-resistance and homeostasis. The red arrow indicates *the pathway that cross-talks with the Hippo core component and enhances the activity of YAP-1*

 The T790M mutation occurs in 50%–60% of cases and increases ATP’s binding affinity to EGFR. This change prevents early-generation EGFR-TKIs from effectively binding to the receptor, leading to drug resistance.^5^ These mutations provided the rationale for developing novel, oral, irreversible third-generation EGFR-TKI, particularly osimertinib (OSI). This ground-breaking therapy initially showed strong efficacy against EGFR-activating mutant cells and T790M-mutated resistant cells.^6^ However eventually, OSI developed resistance. To overcome this challenge, it is crucial to sensitize drug-resistant cancer cells and to enable the development of effective therapies. This could be done by inhibiting an alternative pathway that sensitizes or eliminates resistant cancer cells. On-target and off-target resistance mechanisms are among the many mechanisms of developed resistance that have been identified.^7^ The most recent study demonstrated that Yes-associated protein-1 (YAP-1) expression in NSCLC adenocarcinomas is a potential drug resistance mechanism and inhibiting their co-transcriptional factor can restore the cell’s sensitivity to EGFR inhibitors, including OSI. Recent studies have identified the expression of YAP-1 in NSCLC adenocarcinomas as a potential mechanism of drug resistance. Inhibiting its co-transcriptional factors has been shown to restore cellular sensitivity to EGFR inhibitors, including OSI.^8,9^ The protein YAP-1 is the main transcriptional co-activator of the Hippo pathway. The epithelial-mesenchymal transition is a major resistance mechanism in OSI treatment. It involves a loss of E-cadherin and an increase in vimentin expression, which is further promoted by YAP-1 overexpression. During the progression of resistance, epithelial cells lose their polarity, become motile, and acquire a mesenchymal phenotype.^10^ The essential components of the Hippo pathway consist, of Mammalian sterile 20-like (MST) kinase, large tumor cell suppressor (LATS) kinase, adaptor protein Salvador homolog (SAV1), and Mps one binder kinase activator protein (MOB1). These regulators inhibit the activation of YAP-1, resulting in the suppression of tumor formation.^11^ According to the reports, upregulated YAP-1 promotes cell viability when major driver genes are silenced (Figure 1).^12^ YAP-1 is closely linked to EGFR signaling and is frequently overexpressed in cancer cells resistant to EGFR inhibitors. Studies have shown that inhibiting YAP-1 can restore sensitivity to these inhibitors. While pathways such as PI3K/AKT and MAPK have been extensively studied, the role of YAP-1 in drug resistance and its modulation by natural compounds represents a novel area of research. Targeting YAP-1 addresses a critical gap in overcoming resistance mechanisms, particularly when existing therapies often fall short.

 Thus, combination therapy that inhibits this Hippo pathway was developed to achieve a better outcome.^13,14^ With no new drug exclusively to inhibit chemoresistance in the pipeline, it is highly necessary to search for some alternatives to the anticancer that can substitute or boost the existing anticancer activity. A recent discovery has shown that combining herbs with anticancer medications can effectively reverse the resistance that develops from frequent use of these drugs. This combination specifically targets the oncogenic pathway without harming normal cells.^15^ A major polyphenolic component of green tea (*Camellia sinensis*) is identified as epigallocatechin -3-gallate (EGCG). Many scholarly articles have been published regarding its efficacy in cancer prevention.^16^ It is documented that free radical scavenging and anti-inflammatory activities exert an inhibitory action on cell proliferation.^17^ From the earlier studies, it was found that EGCG was the best natural compound for suppressing Hippo inhibitory protein pathways like EGFR-PI3K-AKT, F-Actin, Wnt, and Rho GTPase.^18,19^ The EGCG may promote YAP-1 degradation by blocking the activity of these proteins, which would decrease the expression of their target genes and reduce the expression of the target genes they bind to. Further, EGCG, a potent antioxidant from green tea, could counteract OSI resistance in NSCLC by disrupting the ROS/HIF-1α/YAP-1 axis, thereby reducing oxidative stress, inhibiting HIF-1α stabilization, and suppressing YAP-1 activity to restore EGFR-TKI sensitivity.

 In the current study, we utilized NSCLC cell lines harboring the EGFR L858R/T790M mutation to evaluate the effects and underlying mechanisms of EGCG in overcoming EGFR-TKI resistance. Furthermore, we demonstrated that EGCG-induced down-regulation of YAP-1 expression represents a promising approach to target chemo-resistance. This strategy could significantly enhance the sensitivity of EGFR-TKIs in EGFR-TKI-resistant NSCLC.

## Materials and Methods

###  Materials

####  Cell lines and reagents 

 The H460, H661, and A549 cell lines of human NSCLC were obtained from the National Centre for Cell Science (NCCS) in Pune, Maharashtra, India. The cells were cultivated in RPMI-1640 medium enriched with 10% fetal bovine serum. The cell cultures were kept in a controlled environment with 95% air and 5% CO_2_ at a temperature of 37°C.

 The compounds EGCG (Catalogue: 989-51-5) and OSI (Catalogue: T10433-5MG) were acquired from Sigma Aldrich/Merck KGaA, located in Darmstadt, Germany. Other substances were obtained from designated vendors. The chemicals Sulforhodamine B (SRB) and 3-(4,5-Dimethylthiazol-2-yl)-2,5-diphenyltetrazolium bromide (MTT), as well as the RPMI medium and fetal bovine serum (FBS), were acquired from Thermo Fisher Scientific Inc. located in Waltham, MA, USA. The TRIzol chemical and Lipofectamine 2000 were acquired from Invitrogen, a company based in Carlsbad, CA, USA. The Clarity Western ECL Substrate and other reagents for western blotting were obtained from Bio-Rad (USA). SYBR-Green master mixture and RT-qPCR analysis reagents were obtained from Takara (Otsu, Japan). All siRNA and Hippo pathway component antibodies are supplied by Santacruz Biotechnology Inc (Santa Cruz, CA, USA).

###  Methods

####  Development of OSI resistant cell lines and transfection 

The OSI-resistant clones (OR) were developed by treating H460 cells following FDA recommendations. Subtoxic doses of OSI (500 nM) were selected. The cells were maintained for two months, with the doses gradually increased every fifteen days until reaching a final concentration of 1.5 µM. Cells treated with OSI were maintained for two months, exhibiting the capability to reinitiate regular growth and proliferation without significant occurrences of cell mortality. Furthermore, the three clones are grown in 96-well plates using the limited dilution method and are kept in RPMI complete medium supplement. Following prolonged cultivation, cell clones exhibiting sustained viability beyond a 14-day period in the presence of 1.5 µM OSI were specifically selected for subsequent assays.^20^

When the cell confluence reached around 90% at the logarithmic growth phase, the 2 × 10^5^ cells were seeded into 6-well plates and transfected, following the manufacturer’s instructions, using lipofectamine 2000 (Invitrogen, NY, USA), with 2.5 µg of short interfering RNAs against YAP-1 and TEAD. The YAP-1-expressing vectors were purchased and transfected according to the manufacturer’s instructions. Gene-edited resistance cells were incubated further for 48 hours. The knockdown of YAP-1 and TEAD was confirmed by western blotting.

####  The sensitivity assay

The responsiveness of the established resistant cell lines to OSI was assessed utilizing the SRB (Sulforhodamine B) cell proliferation test. In summary, 100 μL of H460 cell suspensions were placed in separate wells of a 96-well microtiter plate, with a density of 1 × 10^3^ cells per well. Subsequently, the plates were incubated in a controlled environment of 5% CO_2_ and a temperature of 37 °C. Afterward, the plates were subjected to OSI treatment at dosages ranging from 0.001 to 10 µM. In addition, different concentrations of EGCG were administered to the wells 24 hours after the cells were seeded. After the specified incubation period, the SRB test was conducted according to the instructions provided by the manufacturer.^21^ This assay involves the fixation of cells, followed by staining with sulforhodamine B dye, which binds to cellular proteins. The bound dye is then solubilized, and the absorbance is measured using a spectrophotometer. The resulting absorbance values indicate cell viability and growth inhibition in response to the treatments. Further, the colony formation experiment was performed by inoculating cells into petri plates at a concentration of 100 cells per well and incubating them overnight. The cells were exposed to the specified concentrations of various treatments for a duration of 72 hours and then replaced with new media. Following a week of incubation, the cells were treated with methanol to fix them and then stained with a 0.1% solution of crystal violet for 20 minutes. The visible colonies were then counted and photographed. Each experiment was performed in triplicate.^22^

####  Real-time PCR

Total RNA was extracted from treated and cultured cells using the TRIzol reagent. Complementary DNA (cDNA) was then synthesized through reverse transcription using random hexamers and a cDNA synthesis kit. The resulting cDNA was then subjected to real-time PCR utilizing gene-specific primers in conjunction with the SYBR Green PCR master mix. This process enabled the quantification of mRNA abundance. Specifically, detection was conducted to measure the relative expression of the YAP-1 component.^23^ This method provides insights into the transcriptional activity and regulation of the YAP-1 gene within the cellular context under investigation.

The temperature for qPCR thermocycling was 95 °C for 5 minutes, then 40 cycles of 95 °C for 10 seconds and 60 °C for 34 seconds. Primers specific to each target gene were designed and used in the RT-PCR study. The sequences and details of the primers are listed in Table 1.

**Table 1:** The primers used in RT-qPCR

**Gene **	**Forward primer **	**Reverse primer**
YAP 1	TCCACCAGTGCAGCAGAATA	CACTGGAGCACTCTGACTGA
TEAD 1	TGCAAGGTTTGAGAATGGCC	ATGTTGTGCTCCGTGTTCAC
CTGF	GGCCCAGACCCAACTATGAT	TGGGAGTACGGATGCACTTT
GAPDH	CAAGGCCAACCGCGA GAA	CCCTCGTAGATGGGCACAGT

####  Western blotting 

Western blotting involves lysing cells using a lysis buffer containing specific concentrations of various chemicals such as Tris, NaCl, EDTA, Triton X-100, Na3VO4, NaF, sodium pyrophosphate, sodium β-glycerophosphate, phenylmethylsulfonyl fluoride, aprotinin, leupeptin, and pepstatin A, along with protease inhibitors. Subsequently, the lysate is purified using centrifugation. Protein content quantification is accomplished by employing a BCA test protein estimation kit. In stimulation trials, resistant cells are deprived of nutrients for an extended period and then exposed to the specified concentration and ratio of the test drugs. Equal quantities of protein are placed into the wells of an SDS-PAGE gel, together with molecular weight markers. Next, the protein bands obtained from the gels are transferred onto a nitrocellulose membrane. After the transfer process, a ponceau stain is performed to confirm the accurate transfer of proteins. After thorough washing, the membranes are then exposed to primary antibody solutions that specifically target proteins such as YAP-1, TEAD, and CTGF. The antibodies are usually diluted appropriately and the incubation is typically carried out at a temperature of 4 °C. A secondary antibody (Anti-Mouse HRP 1:30000 TBST) is introduced after the washing step. Finally, the membranes were visualized using labeled antibodies and subsequently quantified.^24^

####  Combination and ratio analysis of EGCG and OSI co-administration

The drug-combination testing utilized Chou’s diagonal constant ratio technique.^25^ The combination analysis was conducted using the equipotent molar ratio of OSI: EGCG, which was determined based on the IC_50_ values obtained from individual treatments of EGCG and OSI in resistant OR cells. The cells were subjected to concentrations equivalent to 0.25, 0.5, 1, 1.5, and 2 times the IC_50_ value of test drug OSI and EGCG while keeping the ratios constant between 1:1 and 1:4 (OSI: EGCG). Afterward, the optimal combination ratio was determined to establish the maximum synergistic effect on resistant cells. The cells were treated simultaneously with different combination ratios (1:1, 1:2, 1:3, and 1:4) with OSI and EGCG. The data analysis was conducted using computational software called Calcusyn, developed by Biosoft in Oxford, UK. This software employs the median effect methodology outlined by Chou to perform multiple drug dose-effect computations. The quantification of the interaction between EGCG and OSI was expressed as a CI. The Chou-Talalay method defines drug interactions using the CI, where CI < 1 indicates synergy (greater combined effect), CI = 1 indicates additivity (effects are equal to the sum), and CI > 1 indicates antagonism (weaker combined effect). The CI was calculated for various levels of Fa (fraction of cells affected, expressed as the percentage of growth inhibition divided by 100).^26^

###  Statistical analysis 

 GraphPad Prism 8.0.2 (GraphPad Software, La Jolla, CA, USA) was used to conduct statistical analysis. The results of each assay were shown as mean ± SD. ANOVA was used to examine the significant difference between the groups, and Dunnet’s post hoc test with *P* < 0.05 is considered statistically significant.^27^

## Results and Discussion

###  EGCG mediated cell proliferation inhibition in NSCLC cells carrying wild-type EGFR

**Figure 2:**
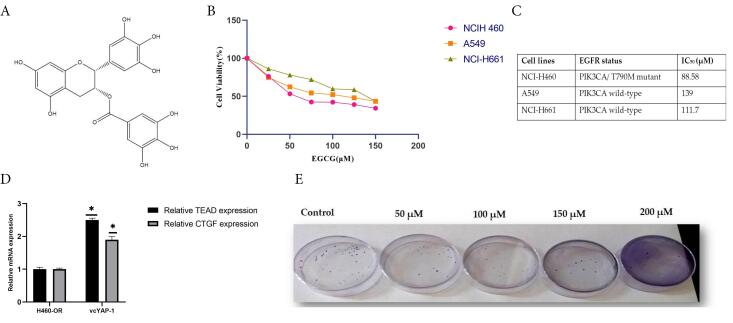
Epigallocatechin-3-gallate (EGCG) inhibited cell proliferation in NSCLC cells, as shown by (A) the chemical structure of EGCG; (B) treatment of H460, A549, and H661 cells with varying concentrations of EGCG (0–150 μM) for 48 hours followed by SRB assay, where cell viability was expressed as a percentage relative to DMSO-treated controls; (C) determination of IC₅₀ values for EGCG in all three NSCLC cell lines using GraphPad Prism version 8.0.2; and (D, E) representative images and quantitative analysis of colony formation in H460 cells treated with specified concentrations of EGCG for 72 hours

Several studies have revealed that EGCG interacts with multiple molecular targets across various signaling pathways, leading to the inhibition of angiogenesis, resistance development, and metastasis, as well as promoting apoptosis and arresting the cancer cell cycle.^28^ In this study, the sulforhodamine B protein assay was employed to assess the outcome of EGCG on NSCLC cell proliferation in three distinct cell lines- A549, H460, and H661 characterized by wild-type EGFR status. According to our results from the cytotoxicity assay, EGCG inhibits cellular proliferation in all three of the examined cell lines in a manner that is dependent on both dose and time. H460 cells harboring the EGFR T790M mutation exhibited greater sensitivity to EGCG treatment compared to H661 and A549 cells, with IC_50_ values of 88.58 ± 0.05 μM, 139 ± 0.1 μM, and 111.7 ± 0.08 μM, respectively, after 48 hours of exposure (Figure 2B, C). A subsequent investigation examined the effect of EGCG on the colony-forming ability of H460 cells (Figure 2D, E). The findings of the study revealed a diminished occurrence of colony formation in H460 cells upon exposure to EGCG.

###  OSI sensitivity in H-460 cell lines and resistant clones using the SRB assay

**Figure 3:**
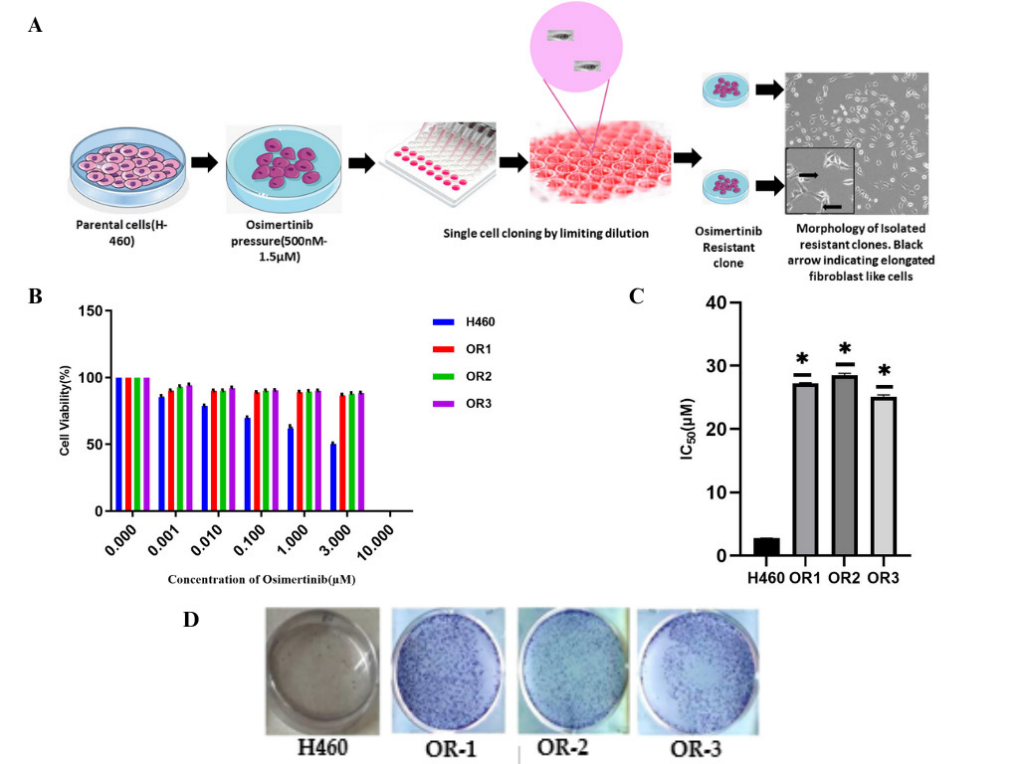
Development of osimertinib-resistant cells and assessment of OSI sensitivity in H460 parental and resistant cells are shown as follows: (A) a schematic representation depicts the development of resistant clones by gradually exposing H460 cells to increasing concentrations of osimertinib (500 nM to 1.5 nM) using a dose-escalation method; (B) SRB assay was performed to evaluate the sensitivity of resistant clones (OR1, OR2, and OR3) and parental H460 cells to osimertinib at various concentrations for 48 hours, and percentage cell viability was determined; (C) IC₅₀ values for OSI were calculated for parental and resistant clones, highlighting the reduced sensitivity in resistant cells; (D) colony formation assay further confirmed the development of resistance by assessing the proliferative potential of cells under OSI treatment (*P < 0.05 considered statistically significant)

 Evolving strategies to treat third-generation OSI resistance focus on understanding the underlying molecular mechanisms, with combination therapies involving other targeted agents being explored to overcome or delay resistance. To explore the mechanism associated with the OSI resistance and to evaluate the effect of their co-treatment with EGCG, H460 resistance cells were developed employing OSI dose escalation followed by limiting dilution to harvest the homogenous resistant clone.^29^ To investigate the in vitro effects of EGCG, drug-resistant cell lines (H460/OR) were established from parental cell lines H460 through a progressive increase in OSI concentration over 4 months (Figure 3A). Each clone was cultured and expanded in a medium containing 1.5 µM OSI concentrations. Subsequently, three clones were meticulously chosen based on their ability to resilience and survive under OSI (OR-1, OR-2, and OR-3) selective pressure for a period exceeding 14 days. High-power microscopy was utilized to examine the morphological characteristics of H460 cells and their OR clones in detail. After receiving OSI therapy, resistant cells formed long, spindle-shaped cells that resembled fibroblast-like cells. The sensitivity to OSI in parental H460 and OR cells was assessed using an SRB assay to determine cell viability. Both cell lines are exposed to OSI at concentrations ranging from 0.001 to 10µM for a duration of 48 h. The resistant clones OR-1, OR-2, and OR-3 exhibited higher IC_50_ values of 27.21 ± 0.10, 28.48 ± 0.34, and 25.12 ± 0.26 µM respectively, compared to the H460 parental cells which had an IC_50_ of 2.74 ± 0.05 µM (Figure 3B). Similar results were obtained with the colony-forming assays, where the resistant cell grew faster than the parenteral cell lines with the OSI treatment (Figure 3C, D).

###  Hippo component proteins YAP-1 confers inherent resistance to the OSI

 Multiple studies demonstrated that Hippo pathway protein YAP-1 induction in NSCLC adenocarcinomas is a potential drug resistance mechanism. The recent study confirms that co-treatment with YAP-1 inhibitors modulates and sensitizes the NSCLC to OSI and other third-generation TKIs.^30,31^

**Figure 4:**
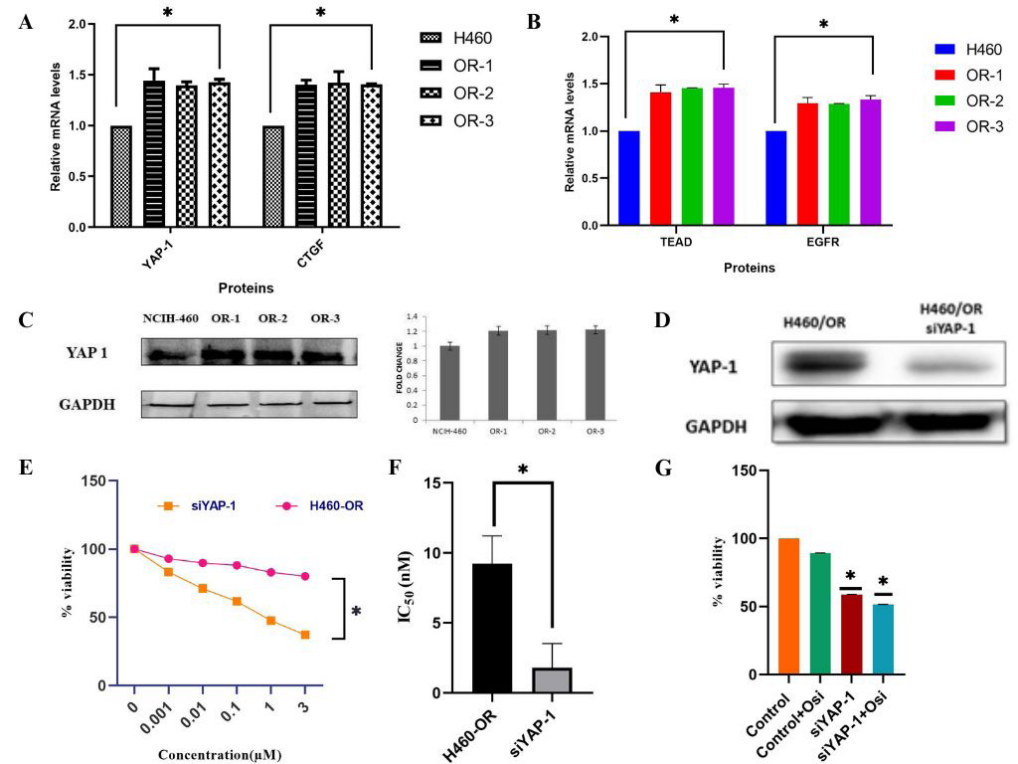
Hippo component YAP-1, TEAD, and CTGF expression in osimertinib resistant cells and knockdown of YAP-1 by siRNA sensitize the resistance cell to OSI (A,B) YAP-1, its downstream target gene CTGF and transcription factor TEAD expression in osimertinib resistant clones were measured by qRT-PCR, the experiments were carried out in triplicate; (C) the overexpression of YAP-1 in the resistant cell was further confirmed by western blotting; (D) the knockdown of YAP-1 by their inhibitor siRNA transfection was evaluated by western blotting; (E,F) after 48 h of treatment with different OSI concentrations, the viability of the YAP-1 siRNA transfected resistant cells was measured. SRB assay was used to measure the IC50 value of YAP-1 depleted resistant cells; (G) the resistance cell function with 5μM OSI, YAP-1 specific siRNA transfected cells, and both were determined by the SRB assay (*P < 0.05 is considered as statistically significant)

 Western blot analysis and quantitative real-time polymerase chain reaction (qRT-PCR) were employed to investigate the potential regulatory role of YAP-1 in conferring OSI resistance in OR clones. It was substantiated that the expression of YAP-1 and its downstream regulators, TEAD, and CTGF was markedly elevated in OR-1, OR-2, and OR-3 cells as compared to their respective parental cell lines. The qRT–PCR study revealed an upregulation of EGFR expression in the resistant cells, suggesting the insensitivity to OSI treatment (Figure 4A). The overexpression of YAP-1 was further established by western blot analysis (Figure 4B). The single clone of OR was selected for the subsequent studies as there was no difference in their gene expression properties. To further elucidate, the impact of YAP-1 in the resistant cells, specific small interfering RNAs (siRNAs), acting as YAP-1 inhibitors, were transfected into the resistant cells. The western blotting analysis confirms inhibited YAP-1 expression in the siRNA vector transfected resistant cells (Figure 4C). The SRB cytotoxicity assay showed that YAP-1 downregulation significantly reduced the IC_50_ of OSI in resistant cells (Figure 4D). To analyze the effect of YAP-1 expression on cell proliferation the SRB assay was conducted and results showed that YAP-1 downregulation significantly inhibited OR cell proliferation. The study also confirmed that treating resistant cells with YAP-1-specific siRNA combined with OSI markedly inhibited the cell function as shown in Figure 4E. These findings imply a correlation between YAP-1 and the progress of acquired resistance to OSI, emphasizing the significant role of YAP-1 overexpression in the development of resistance cells.

###  YAP-1 drives TEAD transcriptional activity in NSCLC

**Figure 5:**
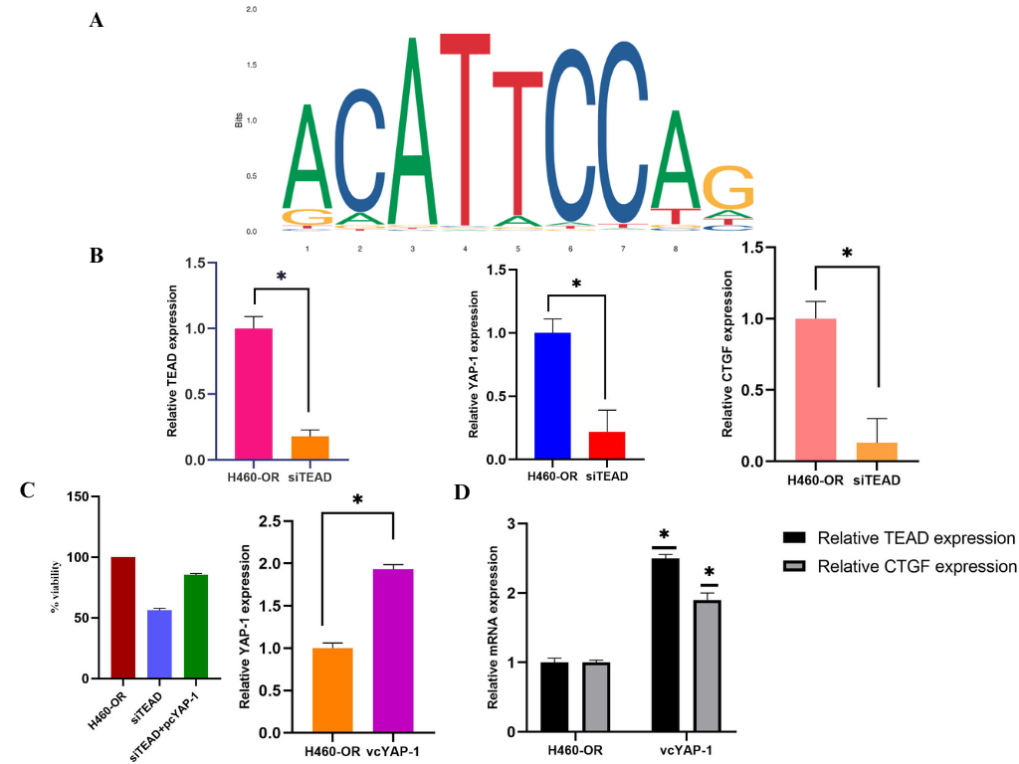
(A) JASPAR prediction of YAP-1 binding site on the sequence of TEAD 1; (B) the OR cells were transfected with siRNA targeting TEAD for 48hr, followed by qRT-PCR experiments were performed in triplicates and relative mRNA expression of TEAD, YAP-1, CTGF was studied; (C, D) The cells were transfected with TEAD si-RNA and YAP-1 expression vector followed by SRB assay, qRT-PCR to analyze mRNA expression of TEAD, CTGF in the presence and absence of vcYAP-1. (At least three independent tests can be illustrated by the findings that were obtained. The information is presented as means ± SD; statistical significance is indicated by *P < 0.05)

 To investigate the resistant mechanism it is essential to identify the downstream elements of transcription factors that are regulated by their association with the transcriptional co-activator YAP-1. A transcription-related factor, a putative oncogene that is increased in human cancer, TEAD mediates YAP-dependent gene activation and growth control.^32^ Being a direct YAP-TEAD target gene, the CTGF is strongly activated by YAP expression.^33^ Using the JASPAR database it was found that the YAP-1 is an upstream transcriptional regulator of TEAD (Figure 5A). Further study is conducted to identify the role of YAP-1-regulated TEAD transcriptional activity in OR cells. The downregulation of YAP-1 also resulted in the inhibition of TEAD and CTGF mRNA expression in the OR cells. The knockdown of TEAD expression inhibits the proliferation of OR cells and reduces YAP-1 and CTGF mRNA expression. The cytotoxic SRB assay and mRNA expression analysis showed that TEAD downregulation significantly reduced the resistant cell proliferation, YAP-1, and CTGF mRNA expression (Figure 4B). Conversely, transfection with the YAP-1 expression vector partially reverses the inhibitory effect on cell proliferation in TEAD down-regulated resistant cells (Figure 5C, D). Overall these data confirm Hippo transducer YAP-1 and its target CTGF drives OSI resistance through their interaction with transcription factor TEA family member protein TEAD-1.

###  Combined treatment of OSI with EGCG exhibit enhanced sensitivity to resistant cells 

**Figure 6:**
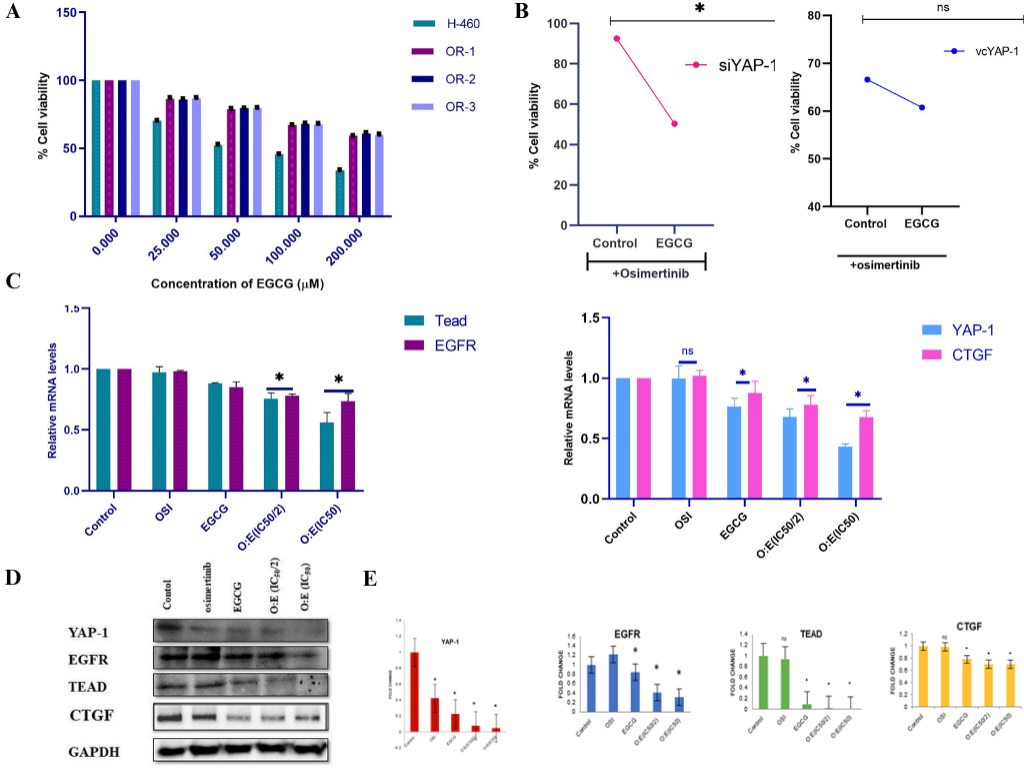
(A) the concentration-dependent effect of EGCG on both resistant clones of OSI and parenteral NSCLC performed by SRB assay; (B) effect of EGCG co-treatment with OSI and mono-therapy of OSI on cell viability of YAP-1 overexpressed and low YAP-1 expressed resistant cells; (C) the mRNA expression of YAP-1, TEAD, CTGF in the resistant cells treated with the indicated concentration and combination of OSI and EGCG by qRT-PCR; (D, E) western blotting analysis and subsequent fold change in the expression of YAP-1/TEAD/CTGF axis proteins in a cell treated with OSI and EGCG

 The SRB test was used to measure the cell viability of each line after they were treated with EGCG at specific doses for 48 hours. The EGCG demonstrated a concentration-dependent decline in cell viability across both parental and clonal cell lines, with IC_50 _values of 102.54 ± 0.23 µM, 225.79 ± 0.30 µM, 237.36 ± 0.17 µM, and 228.71 ± 0.18 µM for H-460, OR-1, OR-2, and OR-3, respectively (Figure 6A). For the synergistic evaluation of EGCG with OSI, resistant cell lines were concurrently exposed to various combination ratios of OSI and EGCG for 48 hours, followed by an assessment of cell viability via the SRB assay. The IC_50_ values of EGCG and OSI in H460 and OR cell line were determined to be 228.71 ± 0.18 µM and 25.12 ± 0.26 µM, respectively. At the starting point, cells were exposed to EGCG and OSI at a molar ratio equal to their corresponding IC_50_ values (EGCG IC_50_/OSI IC_50_). Notably, OSI exhibited approximately a 9-fold greater potency compared to EGCG in resistant cells, leading to the administration of a constant ratio of 1:9 OSI: EGCG concurrently for 48 hours.

 In the context of combination therapy involving chemotherapeutic agents, precise determination of dosage and dose administration ratio is imperative to achieve favorable therapeutic outcomes. The combination of drugs may manifest synergistic, additive, or antagonistic effects contingent upon the combination ratio and administration schedule. The interaction between EGCG and OSI upon simultaneous administration was evaluated employing isobologram analysis, denoted by CI. Utilizing the median-effect principle, CI analysis was conducted to assess the nature of the drug interactions.^34,35^

 Based on the findings, synergistic interactions were evident at lower levels of inhibition ( < 75% inhibition), while additive to antagonistic effects were noted at higher levels of inhibition ( ≥ 90% inhibition). As presented in Table 2, the most pronounced synergistic potency was observed exclusively with the simultaneous treatment regimen at the various concentration ratios of OSI and EGCG. Additive and synergistic cytotoxicity were noted under the simultaneous treatment schedule within combination ratios, specifically ranging from 1:1 to 1:3 molar ratios, with the maximum degree of synergism discovered at the 1:2 ratio. Specifically, at combination ratios of 1:2, synergistic effects were evident across a range from IC_50_ to IC_95_. Furthermore, the cytotoxicity of individual drugs was significantly augmented across various levels of drug effect, particularly when approaching the IC_50 _of each compound when administered in combination. The concurrent administration of EGCG with OSI led to a reduction in the amount of both medications needed to provide 50% inhibition.

**Table 2:** Heat map comparing the effects of OSI and EGCG at various regimens and combination ratios

**Drug combination**	**Combination ratio (µM)**	**Combination Index **			
		**IC**_50_	**IC**_75_	**IC**_90_	**IC**_95_
OSI + EGCG	01:01	1.46 ± 0.33	1.72 ± 0.95	2.09 ± 0.34	2.83 ± 0.25
OSI + EGCG	01:02	0.375 ± 0.68	0.354 ± 0.42	0.334 ± 0.81	0.445 ± 0.54
OSI + EGCG	01:03	1.354 ± 0.13	1.38 ± 0.63	1.412 ± 0.21	3.691 ± 0.27
OSI + EGCG	01:04	1.579 ± 0.67	0.8 ± 0.77	1.14 ± 0.05	5.64 ± 0.45

**Figure:**
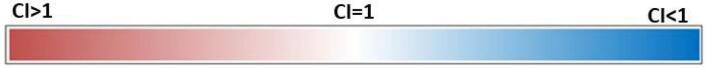 The heat map utilizes a color gradient to represent the synergy index (CI) values, with specific colors indicating different degrees of synergy. In this representation, the red color signifies the lowest CI values, indicating the highest degree of synergism between the drug combinations. White color is employed to denote CI values close to 1, suggesting an additive effect. Conversely, blue color indicates CI values greater than 1, representing a lack of synergism.

###  Combined treatment of OSI with EGCG enhances the anti-tumor activity by inhibiting YAP/TEAD/CTGF axis

 The present investigation was undertaken to clarify the potential anti-cancer effects of EGCG within resistant cells by targeting the YAP/TEAD/CTGF signaling axis. Resistant cell lines exhibiting elevated YAP-1 expression levels and those with diminished YAP-1 expression were subjected to treatment with OSI as a single intervention at a concentration of 25µM and in conjunction with EGCG in a 1:2 molar ratio, throughout 48 hours. Cellular viability was quantified utilizing the SRB assay. The findings demonstrated a statistically significant reduction in cellular viability of YAP-1 overexpressing cell lines following the combined treatment of OSI and EGCG in comparison to OSI monotherapy. Conversely, no variance in cellular viability was observed in OSI-alone-treated low YAP-1 expressing cells compared to co-treatment with EGCG. According to these findings, EGCG and OSI may work in concert to inhibit the proliferation of cancer cells that have high levels of YAP-1 expression (Figure 6B). The subsequent investigation aimed to observe the inhibitory effect of EGCG on the YAP/TEAD/CTGF signaling axis. The cells were exposed to OSI alone and in conjunction with a 1:2 ratio of OSI: EGCG based on their respective concentrations (IC_50_ and IC_50/2_). Protein expression in both control and treated cells was assessed using qRT-PCR and western blotting. The outcome exposed a substantial downregulation of the YAP/TEAD/CTGF axis proteins in resistant cells following the combined therapy. The OSI: EGCG ratio exhibited maximal efficacy at their respective IC_50_ concentrations (Figure 6C, D, E).

 EGFR TKIs are the preferred treatment for patients with NSCLC. OSI is commonly prescribed as an initial treatment for advanced NSCLC cases with prevalent EGFR-sensitizing mutations. This is because it offers better survival benefits and has an acceptable level of toxicity.^36^ However, the emergence of mutations like C797S within the EGFR genes modifies OSI’s binding, leading to resistance and significantly diminishing its clinical effectiveness.^37^ Several natural substances have been scientifically proven to combat resistance to EGFR-TKIs through different methods in preclinical studies. For example, a compound named 35d, a small diarylheptanoid molecule and a curcumin derivative, significantly inhibits tumor recurrence in OR cells by specifically targeting the heat shock protein 70-mediated lysosomal pathway.^38^ Additionally, dioscin has been shown to reduce the expression of SH2 domain-containing phosphatase-2 (SHP2), which plays a role in overcoming resistance to EGFR-TKIs.^39^ Therefore, natural compounds have the potential to be valuable sources for discovering new agents that can effectively overcome resistance to EGFR-TKIs.

 EGCG, a polyphenol found abundantly in green and white tea, has indeed been extensively studied for its potential health benefits, particularly in cancer prevention and treatment. Quercetin, a natural polyphenolic flavonoid, synergistically enhances the therapeutic effect of gefitinib on EGFR-T790M-mutated NSCLC cells by inducing EGFR-T790M degradation through G6PD inhibition and methionine 790 oxidation. This highlights the potential of polyphenol-based combinations, supporting the rationale for testing EGCG with OSI in NSCLC. Recent studies have focused on EGCG’s role in enhancing the effectiveness of chemotherapy while minimizing its side effects. Moreover, EGCG has revealed promise in preventing lung cancer and may have therapeutic potential when used in conjunction with other compounds. It has even been suggested in a recent study that EGCG and gefitinib work in concert to inhibit the proliferation of NSCLC cells that are resistant to the drug. This works in tandem to promote cell death and suppress autophagy. Overall, these findings show the potential of EGCG in cancer treatment and prevention, and its ability to work alongside existing therapies to improve patient outcomes. However, further research is necessary to fully understand its mechanisms of action and optimize its clinical application.^40^

 Recent literature advocates that the dysregulation of YAP/TAZ signaling within the Hippo pathway could significantly contribute to resistance against a range of targeted therapies and chemotherapy. Specifically, the involvement of YAP protein in EGFR mutant NSCLC has been documented. A recent study uncovered that EGFR facilitates the phosphorylation of MOB1, hence, blocking the Hippo pathway and causing YAP/TAZ to be abnormally activated in tumors that have altered EGFR.^41^ Numerous mechanisms contribute to the control of YAP-1 expression levels, encompassing both transcriptional and post-transcriptional processes. Within the Hippo pathway, the TEAD transcription factor family acts as the primary nuclear mediator, with YAP-1 proteins being the predominant co-factors known to activate TEAD.^42^ According to earlier research, anti-CTGF antibodies impeded tumor development and metastasis in mammalian cells, which is the direct target gene of YAP-TEAD.^43^ In our study, we confirmed that YAP-1 activates TEAD and positively regulates the CTGF gene expression in OSI-resistant cells and knockdown of YAP-1/TEAD/CTGF inhibited cell growth and sensitized resistant cells to OSI. Previous research revealed that EGCG exhibited superior efficacy among natural compounds in inhibiting various Hippo inhibitory protein pathways, such as EGFR-PI3K-AKT, F-Actin, Wnt, and Rho GTPase. EGCG’s potential mechanism involves facilitating YAP-1 degradation by impeding the activity of these proteins, consequently reducing the expression of their downstream target genes. Hence, we proposed that the combination of EGCG with OSI could sensitize resistant cells carrying EGFR mutations by targeting the YAP-1/TEAD/CTGF axis. Our findings supported this hypothesis, indicating that EGCG effectively combats observed drug resistance in H460/OR cells. Moreover, besides OSI, it can also efficiently inhibit YAP-1 expression. Further investigations revealed that EGCG inhibits the downstream pathways of YAP-1 by targeting TEAD and CTGF, thereby re-sensitizing OSI-resistant cells to OSI. This suggests that EGCG may exhibit synergy with OSI by targeting the YAP-1/TEAD/CTGF pathway in OSI-resistant cell clones.

 EGCG has shown promising effects in preclinical studies, and its combination with OSI may enhance therapeutic efficacy against EGFR-mutant NSCLC, including overcoming drug resistance. Translating this combination into clinical practice could provide a more effective treatment strategy for patients resistant to OSI alone. While EGCG has demonstrated potential in enhancing the therapeutic effects of chemotherapeutic drugs, there remains a lack of studies investigating its precise mechanism of action and further pharmacological optimization. Moreover, there are no clinical studies yet combining EGCG with chemotherapy, underscoring the need for additional research into its role as an adjuvant therapy.

 The chemopreventive potential of EGCG is highly dependent on its bioavailability and its ability to interact with target tissues. Due to its low lipophilicity, EGCG faces challenges in membrane permeability, particularly across the intestinal epithelium, as it relies on passive diffusion for absorption. To address these bioavailability limitations, nanotechnology applications, such as co-loading EGCG with chemotherapy drugs in nanoparticles, have shown considerable promise. Other strategies, including micronization, lipid-based delivery systems, and co-administration with agents like piperine, could further enhance EGCG absorption. Additionally, transdermal delivery or injectable formulations may offer viable alternatives to improve its bioavailability.

## Conclusion

 In summary, the green tea polyphenol EGCG emerges as a highly promising targeted therapy nutraceutical, demonstrating significant potential for NSCLC patients. Our study elucidates EGCG’s inhibitory action on the overexpressed YAP-1/TEAD/CTGF axis in OSI-resistant clones, synergistically displaying anti-cancer activity by re-sensitising cells to OSI. Consequently, targeting the Hippo component protein YAP-1 represents a promising therapeutic approach against EGFR-TKI resistance. This study lays the groundwork for prospective clinical investigations, aiming to delineate the role of YAP-1 and its downstream regulators in the emergence of chemoresistance. Importantly, this research substantiates, for the first time, the YAP-1/TEAD/CTGF axis as a novel target for the phytoconstituent EGCG, providing a mechanistic insight into how combined treatments reverse acquired OSI resistance.

## Competing Interests

 There is no conflict of interest declared by the authors.

## Ethical Approval

 This study did not involve any experiments on human participants or animals. Ethical approval was not applicable.
